# Influence of decentration of plate-haptic toric intraocular lens on postoperative visual quality

**DOI:** 10.1186/s12886-023-03061-6

**Published:** 2023-07-20

**Authors:** Chunli Diao, Qianqian Lan, Jing Liao, Peng Lu, Zhou Zhou, Lanjian Li, Siming Zeng, Gang Yao, Wei Huang, Qi Chen, Jian Lv, Fen Tang, Min Li, Fan Xu

**Affiliations:** Department of Ophthalmology, Key Laboratory of Eye Health & Guangxi Health Commission Key Laboratory of Ophthalmology and Related Systemic Diseases Artificial Intelligence Screening Technology &Institute of Ophthalmic Diseases, the People’s Hospital of Guangxi Zhuang Autonomous Region & Guangxi, Guangxi Academy of Medical Sciences, Guangxi Zhuang Autonomous Region, Guangxi, China

**Keywords:** Cataract, Toric IOL, Decentration, Visual quality

## Abstract

**Background:**

To evaluate the influence of decentration of plate-haptic toric intraocular lens (IOLs) on visual quality.

**Methods:**

This study enrolled 78 eyes of 78 patients. Patients in group A were implanted with toric IOLs, and patients in group B were implanted with monofocal IOLs. All patients were divided into group A1 and B1 (decentration below 0.3 mm) and group A2 and B2 (decentration above 0.3 mm). The uncorrected distance visual acuity (UDVA), best corrected visual acuity (BCVA), modulation transfer function cutoff (MTF cutoff), objective scatter index (OSI), strehl ratio (SR), optical interference and patients’ satisfaction were measured in different pupils at three months postoperatively. The associations between decentration and visual quality were analyzed by Spearman correlation.

**Results:**

There were no significant differences in UDVA, BCVA, MTF cutoff, OSI, SR, optical interference and patients’ satisfaction among subgroups. The differences in decentration between groups A and B were not statistically significant. In group A2, the total higher order aberrations (tHOAs) at pupil sizes of 3 mm (*P* = 0.046), 5 mm (*P* = 0.014), spherical aberrations at pupil sizes of 3 mm (*P* = 0.011), 4 mm (*P* = 0.014), 5 mm (*P* = 0.000), secondary astigmatism at pupil sizes of 3 mm (*P* = 0.002), 4 mm (*P* = 0.005) were higher than in group B2. Compared to group A1, group A2 had higher spherical aberrations at pupil sizes of 4 mm (*P* = 0.042), 5 mm (*P* = 0.001), 6 mm (*P* = 0.038), secondary astigmatism at pupil sizes of 3 mm (*P* = 0.013), 4 mm (*P* = 0.005), 6 mm (*P* = 0.013). Group B2 has higher coma and secondary astigmatism than group B1 at 6-mm pupil (*P* = 0.014, *P* = 0.045). Significant positive correlations were found between spherical aberrations and the decentration of group A1 and A2 at 6-mm pupils.

**Conclusion:**

The decentration above 0.3 mm negatively affected visual quality due to increased tHOAs, spherical aberrations, coma and secondary astigmatism aberrations, the influence become larger with increasing pupil diameter. And toric IOLs are more affected by decentration than monofocal IOLs.

## Introduction

Astigmatism correction in cataract surgery is a common surgical challenge. Anderson DF et al. [1] systematically reviewed the prevalence of astigmatism and summarized that the proportion of cataract patients with astigmatism more than one diopter (D) ranges from 23 to 47% globally. Residual astigmatism after cataract surgery can lead to poor vision and patient dissatisfaction [2].

The advantages of toric intraocular lens (IOL) for correcting astigmatism have been demonstrated in several studies [3–5]. Multiple previous studies have reported excellent rotational stability of toric IOLs, but few studies have focused on their decentration. Chen X et al. [6] investigated decentration characteristics and related factors of IOLs. Miháltz K et al. [7] and Osawa R et al. [8] compared visual function and decentration of toric IOLs with different haptic designs. Hirnschall N et al. [9] found that decentration contributed to the refractive astigmatic error to some extent. It is critical to ensure a centered position for IOLs in the capsular bag. However, no previous study has divided subgroups according to decentration levels and explored the correlation between varying decentration and visual quality. Therefore, we conducted the current study to assess and compare decentration levels and their impact on visual quality of patients implanted with plate-haptic toric IOLs and monofocal IOLs.

## Materials and methods

### Research design

This is a prospective, nonrandomized controlled clinical study, which complied with the Declaration of Helsinki and was approved by the Ethics Committee of People’s Hospital of Guangxi Zhuang Autonomous Region, China (Ethical Approval Code: KY-LW-202,028). Patients were given detailed explanations of the study protocol and operative complications. They signed an informed consent form to participate in the study and provided permission for the results to be published anonymously.

### Patient selection

Patients who underwent cataract surgery were enrolled at the People’s Hospital of Guangxi Zhuang Autonomous Region between November 2020 and June 2022. Exclusion criteria included a history of vision-limiting ocular diseases or ocular surgeries. In addition, patients with a tilt greater than 7° or the axis rotation of the toric IOL exceeded 10° were excluded.

Patients whose corneal regular astigmatism exceeded 1.0 D were implanted with toric IOLs and included in group A, whereas patients with corneal astigmatism less than 1.0 D were implanted with monofocal IOLs and included in group B. Ale JB et al. [10] reported that a 0.2–0.3 mm decentration are common and clinically unnoticed for any design of IOL. Large magnitude aberrations can be caused by decentrations below 0.5 mm, resulting in significant visual symptoms [11]. In order to investigate whether decentration of 0.3 mm can negatively affect postoperative visual quality, subgroups were divided according to decentration, group A was categorized into group A1 (decentration below 0.3 mm) and group A2 (decentration above 0.3 mm). Group B was categorized as group B1 (decentration below 0.3 mm) and group B2 (decentration above 0.3 mm).

#### Sample size estimation

The sample size is calculated based on the sample size calculation formula (powerandsamplesize.com/). The following assumptions were made for the sample size calculation: type 1 error (alpha) was set at 5%, and power (1-beta) on 0.90. The proportion of cases between the experimental and control groups was set to 1:1. Mean decentration of group A was set to greater than 0.5 mm and mean decentration of group B was set to less than 0.5 mm. Then the sample calculator showed a minimum sample size of 21 patients.

### Intraocular lens

The AT TORBI 709 M IOL (Carl Zeiss Meditec AG, Germany) was used as the toric IOL and the CT ASPHINA 509 M (Carl Zeiss Meditec AG, Germany) was used as the monofocal IOL. All patients’ biometric measurements were taken by the IOL Master 700 (Carl Zeiss Meditec, Jena, Germany) after setting the target refraction to 0 D. The IOL power, alignment axis, and expected residual astigmatism were calculated using the toric IOL calculator program, available at https://zcalc.meditec.zeiss.com/.

### Preoperative examinations

A complete ocular examination was performed on each patient, which included measuring uncorrected visual acuity (UDVA) and best corrected visual acuity (BCVA) and fundus examination. Corneal topographic maps were measured with the iTrace wavefront aberrometer (Tracey Technologies, Houston, TX).

### Surgical technique

With the patients sitting upright in front of a slit-lamp microscope, the skillful assistant precisely marked the axis positions of the toric IOL at the corneal limbus before mydriasis. All operations were performed by experienced surgeons. During the operation, the toric IOL was inserted into the capsular bag and rotated to the planned axis. There were no stitches to close the wound.

### Postoperative examinations

#### Visual acuity and refractive result

Patients were examined at 1 day, 1 week, 1 month, and 3 months postoperatively. UDVA and BCVA were measured at a distance of 5 m with a Standard Logarithmic Visual Acuity chart, then convert to the logarithm of the minimum angle of resolution (LogMAR) vision chart. Refractive error was measured by optometry.

#### Assessment of IOL position

**Fig. 1:**
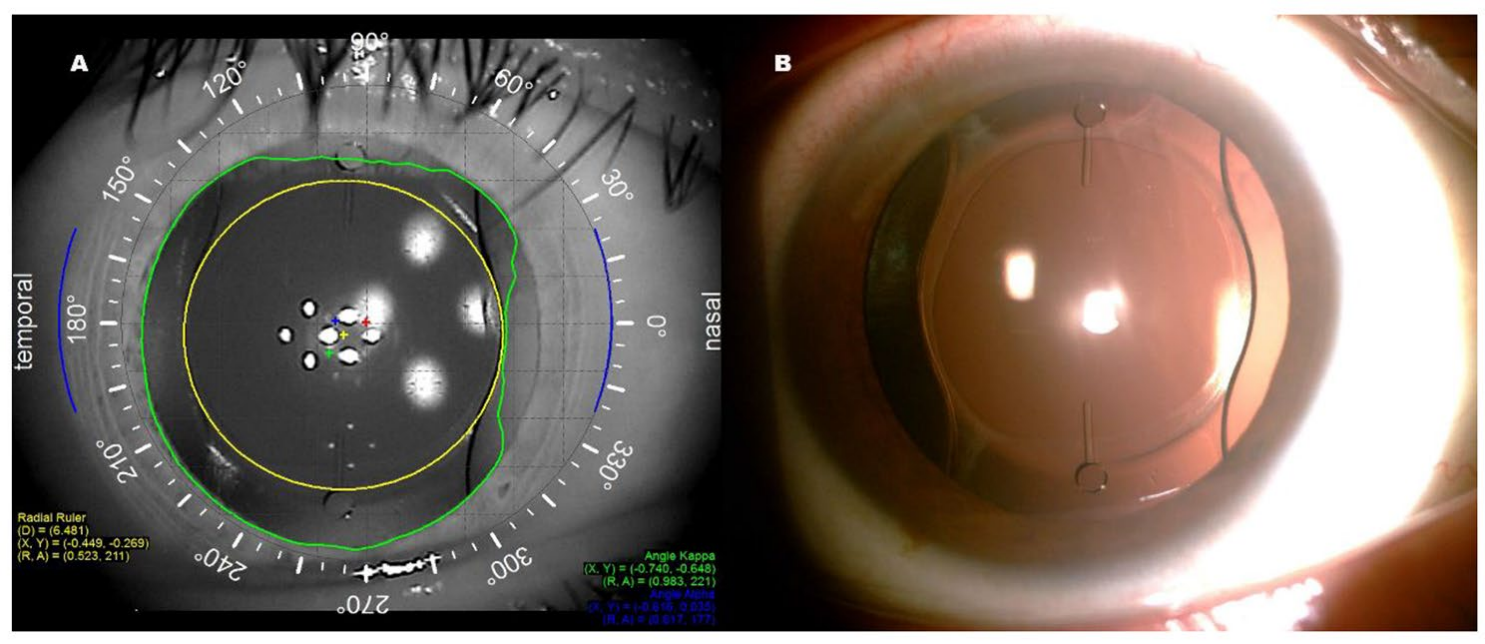
**(A)** Postoperative position of toric IOL with iTrace. The yellow circle was attached to the edge of the toric IOL, and the number on the left was the value of decentration. **(B)** An eye from group A under dilated slit-lamp examination after surgery

The orientation of the toric IOL is highly stable at postoperative 1 day [12], therefore the postoperative results at 3 months can infer the long-term stability of the toric IOL. The decentration was measured by drawing a circle that overlapped the IOL with the iTrace wavefront aberrometer after mydriasis at 3 months postoperatively. (Fig. 1A). During each postoperative visit, patients’ eyes were scanned with a slit lamp for visualizing IOL rotation (Fig. 1B). Patient’s eyes were scanned with six meridians (0, 30, 60, 90, 120, and 150 degrees) to determine the tilt level with IOL Master 700. The measurement method is the same as that of previous research[13].

### Objective visual quality

The Optical Quality Analysis System (OQAS, Visiometrics, Terrassa, Spain) was used to measure objective visual quality including modulation transfer function cutoff (MTF cutoff), objective scatter index (OSI), strehl ratio (SR), OQAS values at contrasts of 100%, 20%, and 9% (OV, the mean of OV 100%, OV 20%, and OV 9%). Since uncorrected spherical and cylindrical errors could directly affect the optical results, the refractive errors of the subjects were completely corrected.

### Aberration measurements

The root mean square (RMS) of whole-eye higher-order aberration (HOA) was measured by using the iTrace wavefront aberrometer. The total higher-order aberrations (tHOAs), coma, trefoil, spherical aberration, and secondary astigmatism were recorded. The entrance pupil scan sizes were 3 millimeters (mm), 4 mm, 5 mm as well as 6  mm respectively.

### Patient satisfaction

[14][15]In this study, patients were interviewed by questionnaire about the frequency and degree of interference from glare, halo, and starburst. During the survey, the researchers explained the definitions of the technical terms in the visual quality questionnaire to the patients to ensure that they all understood the contents of the visual quality questionnaire and to avoid any indiscriminate or missing selections.

### Statistical analysis

IBM SPSS Statistics 25.0 software was used to analyze the data obtained in the study statistically. The normal distribution of numerical data was evaluated with the Shapiro-Wilk test. The Kruskal–Wallis H test was used to compare the postoperative values and Fisher’s exact tests were used to assess the results of a questionnaire. Correlation analysis was conducted by Spearman correlation analysis. For all statistical analysis, data were expressed as mean ± SD. *P* < 0.05 was taken as a significant difference.

## Results

### Patient characteristics and visual acuity

There was no complication occurred in this study. Seven patients were excluded from the analysis due to missing preoperative or postoperative data. Group A included 38 eyes of 38 patients. Group B included 40 eyes of 40 patients. Subject demographics and preoperative and postoperative visual acuity were presented in Table 1.

**Table 1:** Demographics, preoperative and postoperative visual and refraction parameters

Parameter	Group A		Group B		*P-value*
	Group A1	Group A2	Group B1	Group B1	
Sex (Male/ Female)	11/15	7/5	15/12	3/10	
Age	68.38 ± 4.15	69.75 ± 3.11	65.67 ± 6.40	67.08 ± 4.25	0.136
Preoperation					
UDVA (LogMAR)	1.03 ± 0.49	1.07 ± 0.55	1.09 ± 0.57	1.28 ± 0.65	0.691
BCVA (LogMAR)	0.94 ± 0.47	0.80 ± 0.35	0.99 ± 0.50	1.26 ± 0.67	0.391
Corneal astigmatism (D)	-1.72 ± 0.72^b, c^	-1.87 ± 0.75^d, e^	-0.67 ± 0.34^f^	-0.77 ± 0.28^a^	< 0.001^*^
AL (mm)	23.76 ± 1.62	23.22 ± 0.82	23.58 ± 2.66	24.65 ± 2.50	0.114
Postoperative					
UDVA (LogMAR)	0.11 ± 0.12	0.08 ± 0.08	0.11 ± 0.87	0.12 ± 0.12	0.775
BCVA (LogMAR)	0.08 ± 0.11	0.03 ± 0.07	0.05 ± 0.96	0.07 ± 0.13	0.640
Spherical error (D)	-0.07 ± 0.30	-0.02 ± 0.24	-0.08 ± 0.63	-0.13 ± 0.45	0.940
Cylindrical error (D)	-0.43 ± 0.55	-0.52 ± 0.67	-0.27 ± 0.59	-0.29 ± 0.76	0.602
SE (D))	-0.28 ± 0.45	-0.28 ± 0.39	-0.22 ± 0.64	-0.28 ± 0.59	0.887

All data were presented as the mean ± SD. *UDVA* Uncorrected distance visual acuity, *BCVA* Best corrected visual acuity, *LogMAR* Logarithm of the minimum angle of resolution, *SE* Spherical equivalent, *D* Diopter, *AL* Axial length.

^a^*P <* 0.001versus Group A1.

^c^*P <* 0.001, ^d^*P <* 0.001versus Group B1.

^e^*P <* 0.001, ^f^*P <* 0.001versus Group B2.

^*^P < 0.001 among four subgroups.

### Postoperative decentration, rotation and tilt

**Fig. 2:**
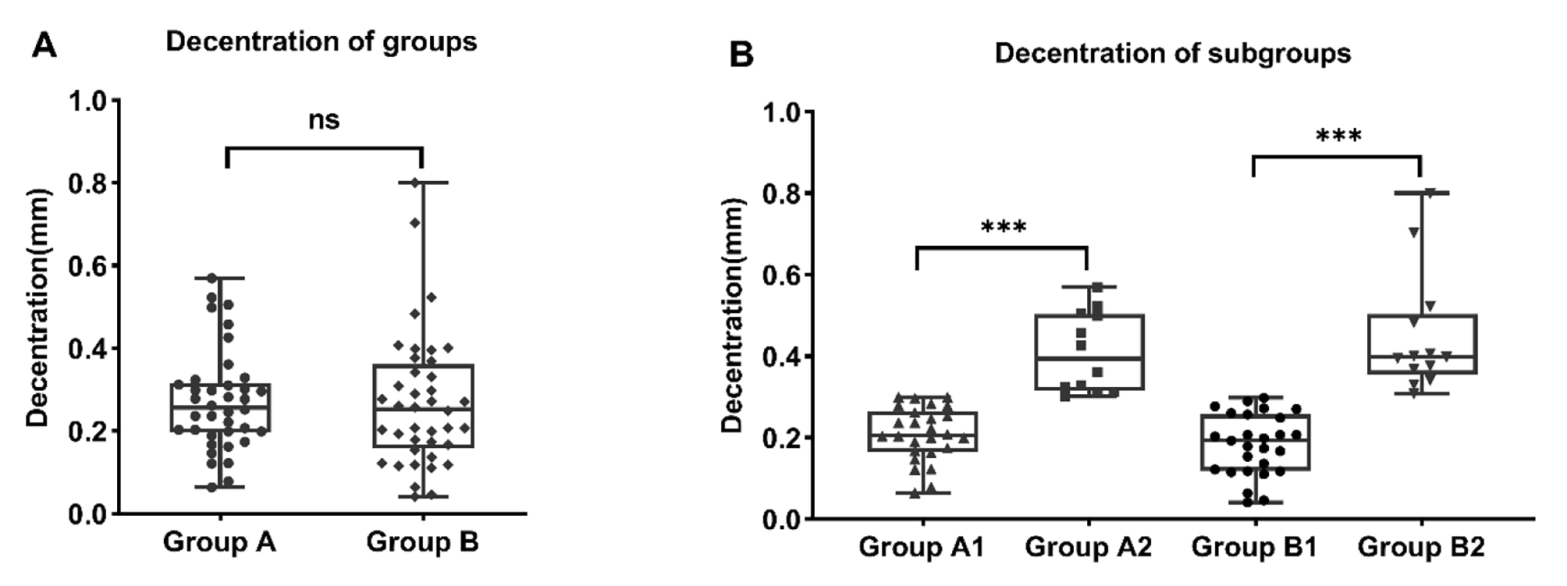
shows the decentration among subgroups. *** means *P <* 0.001

The decentration did not conform to a normal distribution, it was expressed as median and interquartile interval (first quartile; third quartile). Decentration values were 0.26 (0.20, 0.32) mm in group A and 0.25 (0.16, 0.36) mm in group B (Fig. 2). And no statistical significance in decentration was observed between the groups (*P* = 0.598). The mean postoperative rotation was 3.11 ± 2.02 degrees in group A. No tilt of more than 7 degrees in any direction was observed throughout the follow-up period.

### Objective visual quality and aberrations

No statistically significant was observed in objective visual quality characteristics among subgroups (Table 2).

**Table 2:** Postoperative objective visual quality

Parameter	Group A		Group B		*P-value*
	Group A1	Group A2	Group B1	Group B2	
MTF	23.54 ± 10.88	21.51 ± 9.20	26.61 ± 14.09	27.51 ± 14.04	0.658
SR	0.13 ± 0.06	0.11 ± 0.04	0.14 ± 0.06	0.14 ± 0.05	0.242
OSI	2.47 ± 1.96	2.27 ± 1.27	2.04 ± 1.87	2.39 ± 2.34	0.777
OV 100%	0.79 ± 0.35	0.73 ± 0.29	0.87 ± 0.47	0.93 ± 0.46	0.654
OV 20%	0.52 ± 0.26	0.46 ± 0.21	0.62 ± 0.36	0.62 ± 0.32	0.534
OV 9%	0.32 ± 0.17	0.25 ± 0.12	0.35 ± 0.21	0.35 ± 0.17	0.234

All data were presented as the mean ± SD. *MTF* Modulation transfer function cutoff, *SR* Strehl ratio, *OSI* Objective scatter index, *OV 100% OV 20% OV 9%* OQAS values at contrasts of 100%, 20%, and 9%.

**Fig. 3:**
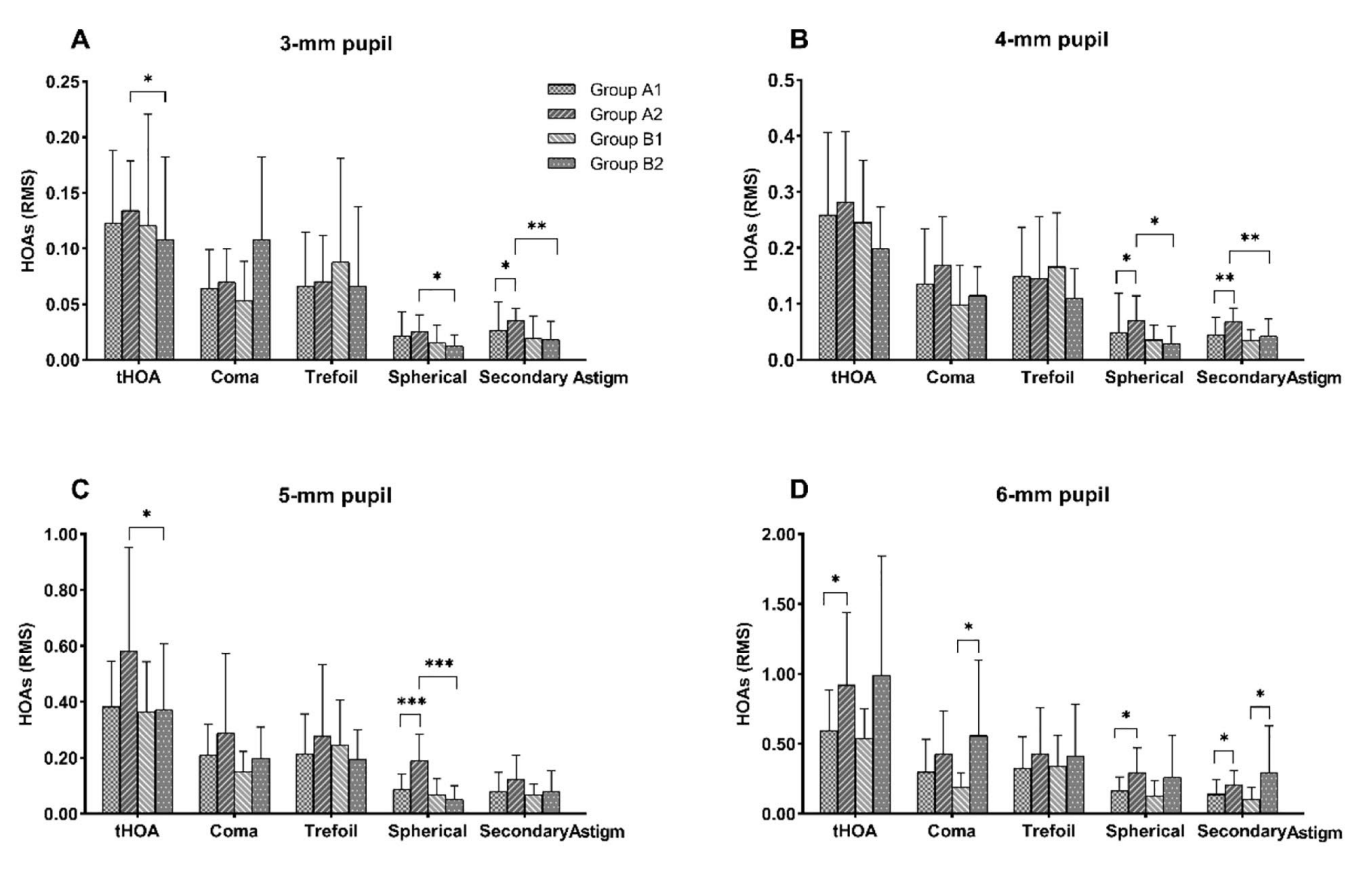
Postoperative ocular higher order aberrations of subgroups with pupil scan sizes of 3 mm, 4 mm, 5 and 6 mm

The results of HOAs were shown in Fig. 3. The tHOAs, spherical aberrations and secondary astigmatisms were significantly higher in group A2 than in group B2 with 3 mm pupil (*P* = 0.046, *P* = 0.011, *P* = 0.002). Group A1 showed lower secondary astigmatism than group A2 (*P* = 0.013; Fig. 3A). The spherical aberrations and secondary astigmatism were higher in group A2 than in group A1 (*P* = 0.042, *P* = 0.005) and group B2 (*P* = 0.014, *P* = 0.005) with 4.0 mm pupil (Fig. 3B). The tHOAs, spherical aberrations were higher in group A2 than in group B2 (*P* = 0.014, *P* = 0.000). Compared to group A1, group A2 had higher spherical aberrations with pupil sizes of 5.0 mm (*P* = 0.001; Fig. 3C). The tHOAs, spherical and secondary astigmatism were higher in A2 than in group A1 (*P* = 0.030, *P* = 0.038, *P* = 0.013). And the coma and secondary astigmatism were smaller in group B1 than in group B2 with 6.0 mm pupil (*P* = 0,014, *P* = 0.045; Fig. 3D).

### Patient satisfaction

**Fig. 4:**
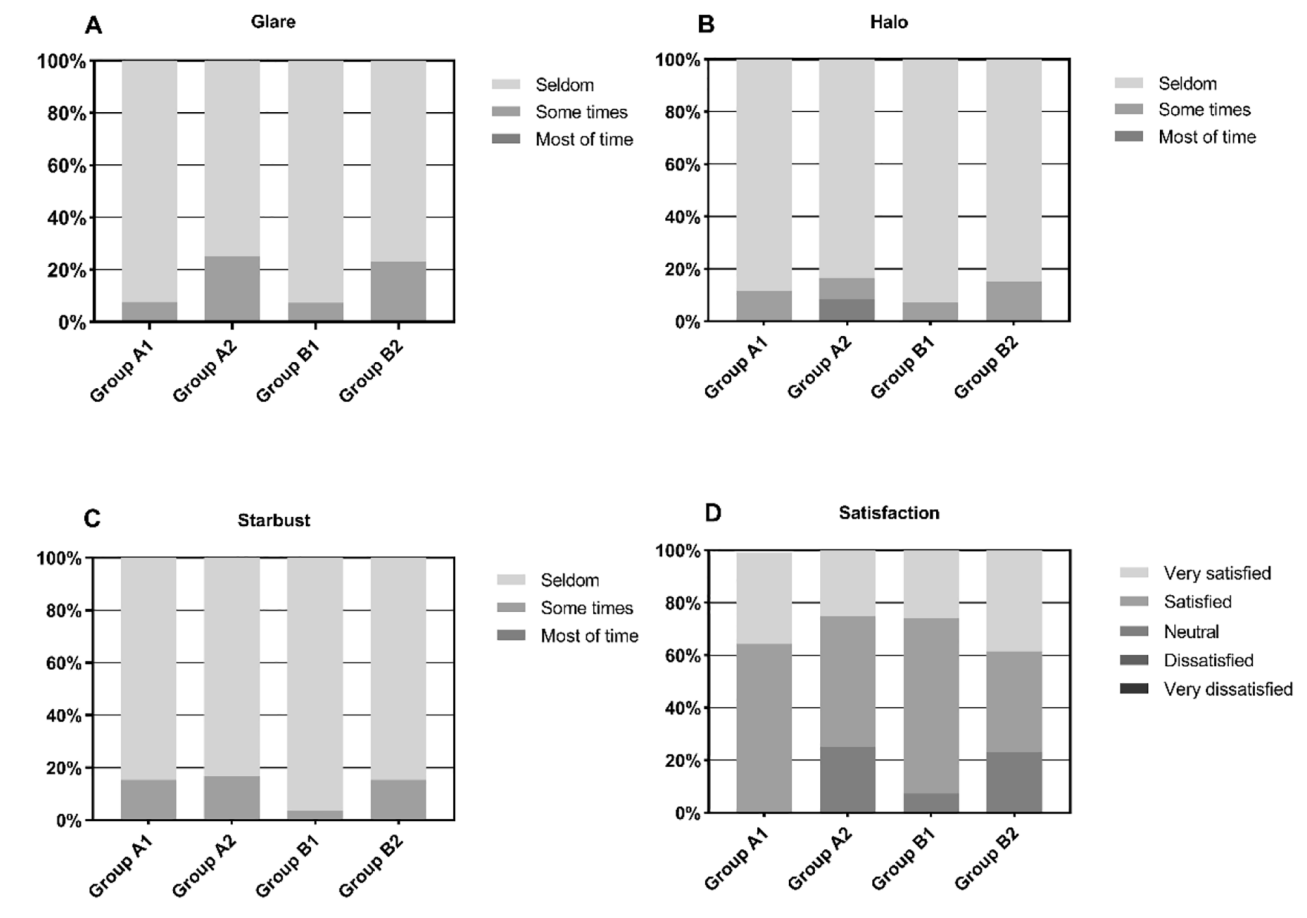
Postoperative responses of subgroups to questionnaire items

Questionnaire responses were reported in Fig. 4. There were no significant differences among subgroups in the incidence of optical interference and overall satisfaction (*P*＞0.05). Patients rarely reported experiencing optical interference phenomena including glare, halo, and starburst. The majority of the patients reported being very satisfied or satisfied with the procedure.

### Correlation analysis

**Fig. 5:**
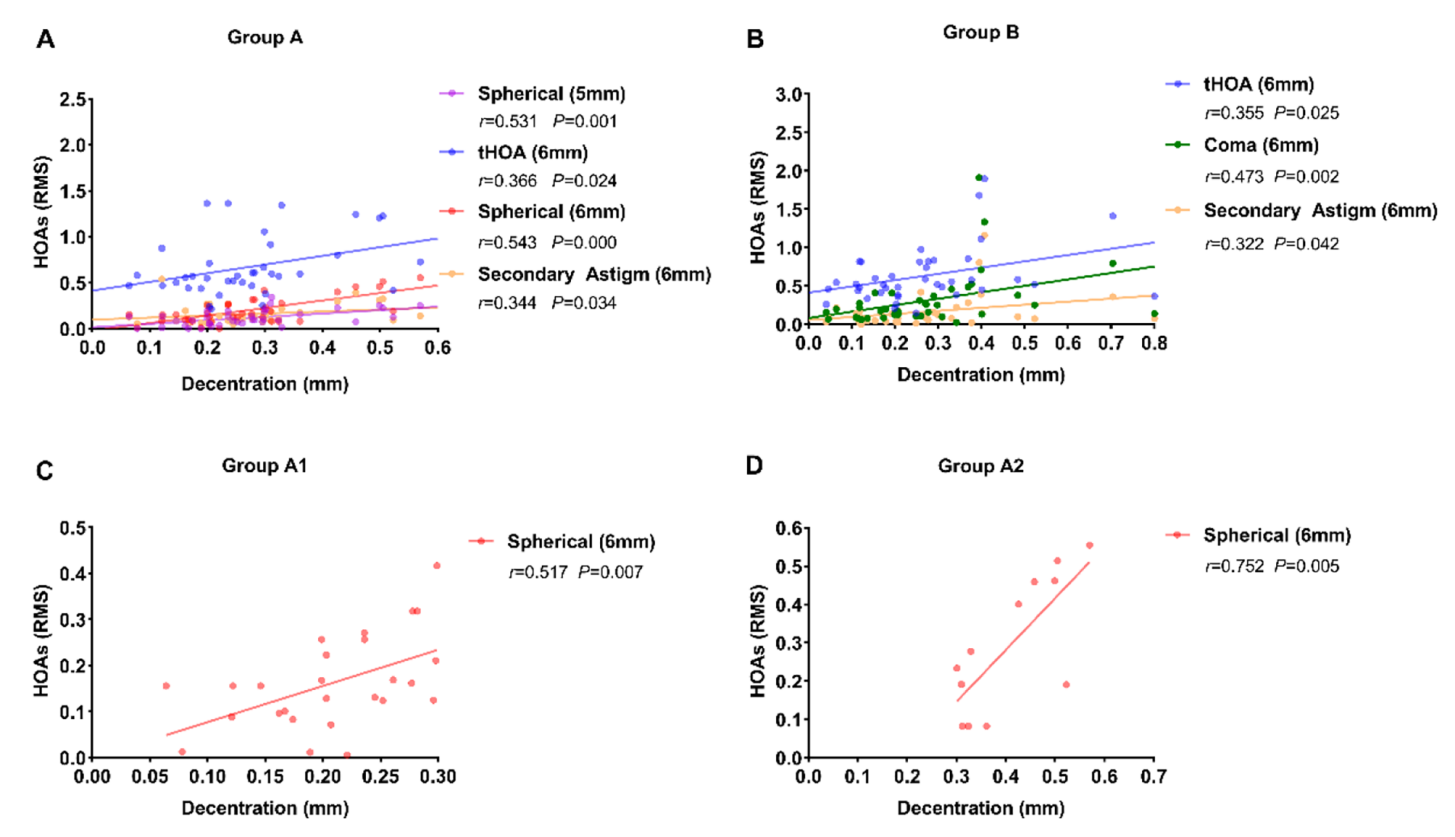
Correlation between decentration and higher order aberrations

The relationships between decentration and HOAs were described in Fig. 5. In group A, decentration was normalized against spherical aberrations (*r* = 0.531, *P* = 0.001) with 5.0 mm pupil, tHOAs (*r* = 0.366, *P* = 0.024), spherical aberrations (*r* = 0.543, *P* = 0.000) and secondary astigmatism (*r* = 0.366, *P* = 0.024) with 6.0 mm pupil (Fig. 5A). In group B, decentration was positively correlated with tHOAs (*r* = 0.355, *P* = 0.025), coma (*r* = 0.473, *P* = 0.002) and secondary astigmatism (*r* = 0.322, *P* = 0.042) with 6.0 mm pupil (Fig. 5B). In group A1, decentration was positively linked to spherical aberrations (*r* = 0.517, *P* = 0.007) with 6.0 mm pupil (Fig. 5C). And we observed a significant positive correlation between decentration and spherical aberrations (*r* = 0.752, *P* = 0.005) with 6.0 mm pupil in group A2 (Fig. 5D).

## Discussion

Previous research has summarized that toric IOLs demonstrated excellent rotational as well as centration stability [16, 17]. IOL decentration may lead to residual cylindrical errors and poor uncorrected visual acuity[18, 19]. Lawu T et al. [20] have compared the effect of decentration and tilt on the optical performance of different aspheric IOL designs in model eyes, the results showed that IOL decentration and tilt increased residual astigmatism and HOAs, and decreased optical quality. Pérez-Vives C et al. [21] evaluate the impact of different decentration levels on the optical quality of three aspheric toric IOLs and indicated that coma aberration increased significantly with IOL decentration. In summary, it is particularly important to ensure that the toric IOL is centered in the capsular bag. However, there are few reports investigating the effect of decentration of the plate-haptic toric IOL on visual quality. In addition, a few studies divided the study subjects into different subgroups based on decentration to further explore the effect of different decentrations on visual quality for different pupil diameters. Therefore, the aim of this study is to compare in the visual quality of plate-haptic toric IOLs and monofocal IOLs in relation to different degrees of decentration at 3-mm, 4-mm, 5-mm, and 6-mm pupils.

According to previous studies, a larger extent of decentration could negatively affect the postoperative optical performance and patients’ satisfaction. Korynta J et al. [22] indicated that astigmatism may be visually significant when decentration exceeds 1.0 mm based on an ophthalmological model. The more complex the optic design of the IOL, the more vulnerable it is to the decentration and tilt [23]. For toric IOLs, decentrations make the result of astigmatism unpredictable. However, the mean decentrations of patients were in the range of 0.2 to 0.3 mm in this study, which was almost consistent with the decentration in previous study [10]. [22][23]And no statistically significant differences were found among the four groups in UDVA and BCVA. This is consistent with previous study, which demonstrated decentration and tilt values after IOL implantations seem to have an effect on HOAs, but not on vision [19].This result implies that the effect of decentration within this range on visual acuity is minor, and probably clinically irrelevant.

Efficacy after cataract surgery can be assessed by optometry results, MTF cutoff, HOAs, patient satisfaction, and so on. The MTF cutoff can be used to quantify the loss of resolution, and the higher the MTF cutoff is, the better the visual quality is reflected [24]. OSI objectively reflects the degree of scattering due to transparency loss in refraction media [25]. According to a previous model eye study, further decreases in MTF cutoff values were observed when decentration was equal to 0.5 and 0.75 mm [26]. In addition,. we found no significant effect of decentration on MTF cutoff, OSI, OV 100%, OV 20%, and OV 9% in either group. The mean decentration of the patients implanted with toric IOLs in this study ranged from 0.2 to 0.3 mm, and the effect on the OQAS index appeared to be minimal. The results reflected that toric IOLs of this study showed favorable tolerance to decentration below 0.3 mm.

IOL position changes such as decentration and tilt can lead to increased HOAs and reduced visual quality [20, 27]. Epping et al. [28] found that decentration led to greater horizontal coma, astigmatism, and defocus aberration at 4.5 mm pupil. Ning Y et al. [29] suggested that coma changes significantly only when the decentration was above 0.2 mm and the tilt was above 2.8º [30]. In case of decentration greater than 0.5 mm, aspheric IOLs lose their advantages [11]. When decentration was equal or above 0.9 mm, VA did not reach 0.2 LogMAR in any eyes [31]. With IOLs decentered by 1.0 mm, the contrast reduction rates of transitional conic toric IOL, bitoric IOL, posterior toric surface IOL, and anterior toric surface IOL were 5.1%, 3.1%, 12.2%, and 15.8%, respectively [32]. In this study, significant differences in HOAs were observed with respect to different subgroups. The HOAs of the subgroup with high decentration were greater than that of the subgroup with low decentration. Group A1 had statistically significantly lower spherical aberrations than group A2 at 3-mm, 4-mm, and 5-mm pupils, which indicated that decentration of toric IOLs mainly affected spherical aberration. In summary of the above results of this study and of previous reports, a decentration exceeding 0.30 mm can result in increased HOAs and reduced visual quality. Toric IOLs with good position and well-centered could decrease HOAs and improve optical performance.

Previous studies have shown that high coma often leads to double vision [33], and spherical aberrations are associated with glare and halo [34]. In this study, noobody complained of photic phenomena frequently, and most patients were very satisfied or satisfied with the postoperative results after 3 months postoperatively. And there was no difference in satisfaction and various types of photic phenomena within the four subgroups. Therefore, we summarized that the effects of decentration on photic phenomena and patients’ satisfaction were so small that it might be clinically insignificant. In addition, we had to consider that these results were directly related to the small number of subjects.

In our research, we detected that spherical aberrations were getting larger with increasing decentration of group A1 and group A2 at 6-mm pupils. When the pupil gets larger at night, so do the spherical aberrations. Thus, a high level of decentration may contribute to the elevated glare and halo risk in the evening. We summarized previous studies and our research find that the HOAs after toric IOL implantation may be increased when the decentration is more than 0.3 mm, which could decrease the visual quality of patients. Therefore, the postoperative decentration should be monitored after the implantation of toric IOLs.

There have been many advances in methods of measuring IOL decentration and tilt recently. In previous studies, tilt and decentration were measured by the Purkinje imaging technique, Scheimpflug imaging, ultrasound biomicroscopy, and anterior segment OCT [11]. In our study, we used the iTrace wavefront aberrometer for decentration and aberration measurements. In order to meet the increasing visual requirements of patients, the iTrace wavefront aberrometer is a good device for further research on decentration and visual quality in hospitals without the above ophthalmological examination devices, which is beneficial to promote the development of refractive cataract surgery.

The current studies demonstrate that the position of the preoperative crystalline lens and AL were the crucial influencing factors of IOL decentration and tilt [35]. Moreover, capsulorhexis and capsular fibrosis have an effect on IOL decentration. Severe eccentric capsulorhexis predisposes patients to greater IOL decentration [36]. Capsular stability plays an important role in the optical performance of IOLs [37]. In conclusion, it is important to select patients rationally, examine patients delicately, mark axial positions accurately and perform surgical operations skillfully to reduce postoperative decentration of toric IOL.

There are some limitations of this study. Firstly, the results were directly affected by the small sample size of patients, especially the small number of patients with high decentration. Secondly, patients were followed for 3 months without assessment of clinical outcomes over a longer period. In future studies, the follow-up period should be extended., In addition, there was an inevitable error in the results because the decentration was measured using iTrace by manually drawing a circle until the circle coincided with the edge of IOLs’ optical area. In order to reduce the errors, the inspector measured decentration multiple times and took the average value in this study.

In conclusions, the toric and monofocal IOLs in this study are capable of showing a low degree of decentration and providing excellent visual quality and patient satisfaction. And eyes are tolerant to the mild decentration below 0.3 mm of toric IOLs. Compared with monofocal IOLs, the decentration above 0.3 mm of toric IOLs have a greater negative effect on the visual quality due to increased tHOAs, spherical aberrations, coma and secondary astigmatism aberrations, the influence become larger with increasing pupil diameter.

## Data Availability

The datasets generated and analyzed during the current study are not publicly available due to ethical restrictions but are available from the corresponding author on reasonable request. The data that support the findings of this study are available from the corresponding author, Fan Xu, upon reasonable request.

## Abbreviations

IOL: Intraocular len
UDVA: Uncorrected distance visual acuity
BCVA: Best corrected visual acuity
LogMAR: Logarithm of the minimum angle of resolution
OQAS: Optical Quality Analysis System
MTF cutoff: Modulation transfer function cutoff
OSI: Objective scatter index
SR: Strehl ratio
OV100%, OV20%, and OV9%: Mean OQAS values 100%, 20% and 9%
HOAs: Higher order aberrations
D: Diopter
AL: Axial length
SE: Spherical equivalent
RMS: The root mean square
